# Asymmetric encoder-decoder U-net with hybrid attention and residual dilated convolution for brain tumor segmentation

**DOI:** 10.3389/fnins.2026.1999080

**Published:** 2026-10-28

**Authors:** Xiaoshuai Cao, Xige Song, Jincan Zhang, Wenna Chen, Ganqin Du

**Affiliations:** 1 The First Affiliated Hospital, and College of Clinical Medicine of Henan University of Science and Technology, Luoyang, China; 2 College of Information Engineering, Henan University of Science and Technology, Luoyang, China

**Keywords:** brain tumor segmentation, hybrid channel-spatial attention, magnetic resonance imaging, medical image analysis, multi-scale feature extraction

## Abstract

**Inroduction:**

The accurate and automatic segmentation of brain tumors from magnetic resonance imaging is regarded as essential for clinical diagnosis and surgical planning. Nevertheless, the concurrent extraction of both local fine details and global contextual cues remains challenging, primarily due to ambiguous tumor margins and marked disparities in tumor dimensions.

**Methods:**

To address these issues, a novel HCSA-RMSDC U-Net is designed in this work, which is characterized by an asymmetric architecture with a computationally heavy encoder and a lightweight decoder. Specifically, a hybrid channel–spatial attention (HCSA) module is incorporated into the encoder, wherein axial decomposed convolution is utilized to capture directional spatial information, thereby enhancing spatial feature representation for tumor segmentation. Concurrently, a residual multi-scale dilated convolution (RMSDC) module is deployed in the bottleneck layer to capture semantic information across varying scales without increasing the spatial resolution.

**Results:**

The proposed model was evaluated on the publicly available BRISC 2025 and Figshare datasets, achieving Dice coefficients of 87.39% and 82.84%, respectively

**Discussion:**

These findings support the complementary roles of the HCSA and RMSDC mechanisms in enhancing feature representation and multi-scale contextual modeling. Overall, the proposed framework provides a fully automated approach for brain tumor segmentation.

## 1 Introduction

Gliomas are recognized as the most prevalent and lethal primary malignant tumors of the central nervous system (Price et al., 2024). According to statistics from the Central Brain Tumor Registry of the United States, approximately 30% of all brain tumors are accounted for by gliomas, and a median survival of only 12 to 15 months is observed in patients with glioblastoma (Ostrom et al., 2022). In clinical practice, magnetic resonance imaging (MRI) has been established as the preferred imaging modality for brain tumor diagnosis, surgical planning, and treatment evaluation, owing to its superior soft-tissue contrast (Abidin et al., 2024). However, accurate tumor delineation from MRI is rendered highly challenging by several factors, including blurred tumor boundaries, substantial overlap in gray-level distributions between tumors and normal tissues, and considerable inter-patient variability in tumor size, shape, and location (Zhang et al., 2022). Traditional segmentation methods, which are predominantly based on manual annotation or semi-automated image processing techniques such as region growing, level sets, and active contour models, are not only time-consuming and labor-intensive but also heavily dependent on operators’ prior knowledge and experience, thereby limiting their feasibility for meeting growing clinical demands (Gunasekara et al., 2021). Therefore, the development of automated brain tumor segmentation methods that enable accurate and efficient delineation of tumor regions from MRI images remains an important research direction (Karim et al., 2024).

Deep learning, particularly U-Net-based encoder-decoder architectures, has become a widely adopted paradigm for medical image segmentation (Alzubaidi et al., 2021; Ronneberger et al., 2015). Subsequent studies have extended this framework through three-dimensional modeling, structural redesign, attention mechanisms, multi-scale feature extraction, and Transformer-based contextual modeling, as represented by V-Net (Milletari et al., 2016), UNet++ (Zhou et al., 2018), Attention U-Net (Oktay et al., 2018), DeepLab (Chen et al., 2018), MultiResUNet (Ibtehaz and Rahman, 2020), TransUNet (Chen et al., 2024), and Swin-UNet (Cao et al., 2023). Despite these advances, brain tumor segmentation remains challenging because accurate delineation requires effective feature discrimination together with multi-scale contextual modeling, particularly for tumors with large variations in size and morphology.

Despite the notable progress that has been achieved by the aforementioned approaches, several critical limitations are still observed when these methods are applied to brain tumor segmentation. In several attention-enhanced U-Net variants, attention mechanisms are introduced primarily at skip connections or feature-fusion stages, whereas progressive attention-based refinement within the encoder has received comparatively less emphasis (Khodadadi and Shakoor, 2025; Sanghyun et al., 2018). Given that the encoder serves as the starting point of the entire feature hierarchy, insufficient feature selection at early stages may allow irrelevant background responses to propagate to subsequent layers, potentially weakening tumor-related feature discrimination (Abrar et al., 2025). With regard to multi-scale methods, including DeepLab and PSPNet, although dilated convolutions and pyramid pooling are effective in enlarging the receptive field, these modules are often introduced as standalone substitutes and are not tightly integrated with the U-Net encoder-decoder backbone (Sulaiman et al., 2024). Furthermore, a symmetric architecture is adopted by most existing methods, in which modules of comparable complexity are deployed in both the encoder and decoder; however, the fundamental asymmetry in information flow is neglected—the encoder is primarily responsible for hierarchical feature extraction and discrimination, whereas the decoder mainly performs feature fusion and spatial reconstruction. This functional difference motivates investigating whether enhancement capacity can be allocated differently across the two pathways (Jiang et al., 2024). Applying comparable enhancement modules to both pathways may introduce additional computational cost, motivating a more stage-specific allocation of feature-enhancement capacity.

In order to overcome the aforementioned limitations, a novel asymmetric encoder–decoder U-Net architecture is designed. Unlike traditional symmetric designs, in which the encoder and decoder are treated equally, the designed network is deliberately designed with a “heavy encoder, light decoder” philosophy, whereby computational resources are concentrated precisely where they are most needed. This design is guided by two core principles. First, for encoder-side discriminative enhancement, a hybrid channel–spatial attention (HCSA) module is embedded within each encoder double-convolution block. In contrast to attention mechanisms based on global pooling, by which spatial information is uniformly compressed, depthwise strip convolutions along the horizontal and vertical directions are employed in the designed HCSA to capture anisotropic long-range dependencies. Since tumor boundaries and infiltrative regions often exhibit non-uniform and direction-dependent spatial structures, direction-sensitive spatial context modeling is enhanced through the introduction of strip convolutions in both directions, with low computational overhead being incurred. The channel and spatial branches are dynamically combined via learnable adaptive fusion weights, by which the encoder is enabled to progressively strengthen feature responses related to tumor regions during feature extraction. Second, for bottleneck multi-scale receptive field expansion, a residual multi-scale dilated convolution (RMSDC) module is introduced at the bottleneck to enhance multi-scale contextual feature representation. This module is composed of four parallel branches: one identity branch, by which local semantic information is preserved, and three 3 × 3 dilated convolution branches with dilation rates of 1, 2, and 3, respectively, through which tumor structures at different scales—ranging from small focal lesions to large diffuse areas that may occupy an entire lobe—are captured. The outputs of all branches are concatenated and fused by a 1×1 convolution. The fused multi-scale features are modulated by a learnable residual coefficient before being added to the module input, allowing the contribution of the newly introduced residual features to be adaptively regulated during optimization. Crucially, only standard upsampling convolutions are retained in the decoder, without additional attention or multi-scale modules being introduced, thus embodying the “light decoder” strategy, by which the benefits gained from the enhanced encoder and bottleneck are preserved while redundant computation is minimized.

It should be emphasized that the contribution of this work does not arise from introducing channel attention, directional convolution, or dilated convolution as individually new operations. Rather, the novelty lies in their task-oriented organization and stage-specific integration within an asymmetric encoder–decoder architecture. Specifically, HCSA is used for progressive feature refinement in the encoder, RMSDC is employed for multi-scale contextual aggregation at the bottleneck, and the decoder is intentionally kept lightweight. This design enables the two modules to perform complementary functions at different stages of the network.

On the basis of the aforementioned strategy, the proposed asymmetric HCSA-RMSDC U-Net combines encoder-level feature enhancement with bottleneck-level multi-scale context aggregation while retaining the concise encoder-decoder framework of the standard U-Net. The main contributions of this paper are summarized as follows:

1. A hybrid channel–spatial attention (HCSA) module is designed, in which strip-wise decomposed convolutions are employed for efficient long-range dependency modeling at the encoder stage, thereby enhancing directional spatial context modeling and encoder-side feature representation.
2. A residual multi-scale dilated convolution (RMSDC) module is designed, in which multi-scale contextual information is captured at the bottleneck with low computational cost, and the network’s adaptability to tumors of varying sizes is thereby improved.
3. An asymmetric encoder–decoder HCSA-RMSDC U-Net is constructed on the basis of the standard U-Net backbone, which integrates encoder feature discrimination with bottleneck multi-scale aggregation in a stage-specific manner. Extensive experiments are conducted on brain tumor MRI datasets, and the proposed method achieves higher segmentation accuracy than several mainstream approaches. The individual and combined contributions of the HCSA and RMSDC modules are therefore evaluated through these experiments.

## 2 Related work

Brain tumor segmentation has progressively evolved from conventional convolutional encoder-decoder networks toward architectures incorporating attention-based feature refinement, multi-scale contextual modeling, and more efficient global representation. Recent brain tumor segmentation studies have explored attention-enhanced convolutional U-Net architectures, efficient encoder-decoder networks, and hybrid state-space modeling (Sun and Qin, 2026). Recent reviews have also summarized the growing importance of feature representation, contextual modeling, and architectural optimization in MRI brain tumor segmentation (Bibi et al., 2025; Yadav and Kolekar, 2026). Accordingly, this section focuses on three lines of research most closely related to the proposed method: U-Net-based structural optimization, attention-based feature refinement, and multi-scale contextual modeling with stage-specific architectural design.

### 2.1 U-net-based structural optimization and brain tumor segmentation

Following the development of fully convolutional networks for end-to-end pixel-level segmentation (Shelhamer et al., 2015), U-Net-based encoder-decoder architectures have been extensively extended through modifications to feature connectivity, network depth, and information fusion. ParaU-Net, for example, employs a parallel encoding strategy to improve feature representation (Lu et al., 2024), while recent reviews have summarized broader developments and emerging trends in U-Net architectures for medical image segmentation (Hossen et al., 2026). Three-dimensional spatial modeling has also been explored through 3D U-Net (Çiçek et al., 2016), whereas dense feature connectivity has been incorporated into DenseUNet to facilitate feature propagation (Cao et al., 2020). More recently, learnable skip connections have been investigated to reduce the semantic discrepancy between encoder and decoder features (Wang et al., 2024), and multi-scale convolution-attention mechanisms have been introduced to jointly improve contextual representation and feature selection (Pan et al., 2025). These developments demonstrate that the performance of U-shaped architectures depends not only on the backbone itself but also on how feature-enhancement mechanisms are distributed across different network stages. In particular, encoder, bottleneck, skip-connection, and decoder stages serve different information-processing functions. These developments motivate examining whether feature-enhancement capacity can be allocated according to the functional roles of different encoder-decoder stages rather than being increased uniformly across the network.

The proposed architecture follows this stage-dependent perspective. Rather than repeatedly deploying enhancement modules throughout the entire encoder-decoder network, HCSA is concentrated in the encoder for progressive feature refinement, RMSDC is introduced only at the bottleneck for multi-scale contextual aggregation, and the decoder is deliberately kept lightweight. This organization is intended to allocate feature-enhancement capacity according to the functional role of each network stage.

### 2.2 Attention-based feature refinement

Attention mechanisms have become an important approach for improving feature selection in medical image segmentation. Recent reviews have summarized the development of attention mechanisms from conventional spatial and channel recalibration to more advanced contextual and state-space modeling strategies (Zhang et al., 2025; Sun et al., 2026). Classical approaches include channel-wise feature recalibration through the Squeeze-and-Excitation mechanism (Hu et al., 2018) and spatially selective attention gating for medical image segmentation (Schlemper et al., 2019). These methods demonstrate that explicitly weighting task-relevant features can improve the utilization of intermediate representations.

Subsequent studies have expanded the spatial modeling range of attention mechanisms. Axial attention models long-range dependencies separately along spatial axes (Wang et al., 2020), while cross-axis attention has been explored in medical image segmentation to integrate directional and multi-scale spatial information (Shao et al., 2025). More recent architectures have further investigated asymmetric attention-enhanced encoder-decoder design (Su et al., 2026), adaptive spatial-channel feature enhancement and fusion (Zhao et al., 2026), multi-granularity attention (Wang and Lim, 2025), and lightweight multi-scale attention mechanisms (Wang et al., 2024). Collectively, these developments show a transition from isolated feature recalibration toward more structured integration of channel, spatial, and contextual information.

The proposed HCSA module differs from these approaches in both its internal organization and its network placement. Unlike attention mechanisms that are primarily applied to skip connections or decoder-side feature fusion, HCSA is embedded directly within the encoder double-convolution blocks so that feature refinement is performed progressively during encoding. In addition, channel and spatial representations are constructed in parallel rather than sequentially. The spatial branch employs horizontal and vertical depthwise strip convolutions to capture directional contextual information, while the relative contributions of the channel and spatial branches are adaptively regulated through learnable fusion coefficients. Thus, the distinguishing characteristic of HCSA lies in the combination of parallel channel-spatial modeling, directional convolutional context extraction, learnable fusion, and encoder-specific deployment.

### 2.3 Multi-scale context modeling and stage-specific design

Multi-scale representation is particularly important in medical image segmentation because target regions may exhibit substantial variations in size, morphology, and spatial extent (Yang et al., 2026). Representative strategies for enlarging the contextual modeling range include feature-pyramid structures (Lin et al., 2017), pyramid pooling (Zhao et al., 2017), and dilated convolution, which has been widely discussed in medical image segmentation research (Wang et al., 2022). Parallel convolutions with different receptive fields, as exemplified by multi-kernel architectures such as Inception (Szegedy et al., 2015), provide another means of extracting information at multiple spatial scales.

The importance of multi-scale representation is especially relevant to glioma and brain tumor segmentation, where substantial heterogeneity in tumor size and morphology is commonly encountered (Shoushtari et al., 2025). DeepMedic provides an early example of multi-path multi-scale processing for brain lesion segmentation, combining information obtained from different receptive ranges (Kamnitsas et al., 2017). More recent medical image segmentation approaches have integrated multi-scale dependency modeling with attention-guided enhancement (Li M. et al., 2025) and state-space-based feature modeling (Li D. et al., 2025). Recent brain tumor-specific work has also combined multi-scale feature extraction with attention-enhanced U-Net architectures to improve contextual representation across different resolutions (Nisha et al., 2025). Although these methods demonstrate the value of multi-scale contextual information, the location and manner in which multi-scale enhancement is introduced remain important architectural considerations. Reviews of U-Net variants have emphasized that different encoder-decoder stages perform distinct functions, including hierarchical feature extraction, semantic aggregation, feature fusion, and spatial reconstruction (Siddique et al., 2021). Brain tumor segmentation has also been investigated using cascaded densely connected U-Net architectures to enhance volumetric feature representation (Ghaffari et al., 2021). Together, these studies motivate further investigation of where multi-scale enhancement is most appropriately introduced within an encoder-decoder architecture, rather than assuming uniform deployment across all stages.

The proposed RMSDC module is therefore restricted to the bottleneck, where high-level semantic features are available and the spatial resolution has already been reduced. RMSDC combines an identity-preserving branch with three compact dilated-convolution branches using dilation rates of 1, 2, and 3. The resulting multi-scale features are fused and injected into the original bottleneck representation through channel-wise learnable residual scaling. Unlike approaches that repeatedly introduce multi-scale processing throughout the network, RMSDC performs contextual aggregation only at the bottleneck while preserving a lightweight decoder.

Overall, existing studies have demonstrated the importance of structural optimization, attention-based feature refinement, and multi-scale contextual modeling for medical image and brain tumor segmentation. The proposed HCSA-RMSDC U-Net differs mainly in the stage-specific allocation of these mechanisms: HCSA performs progressive channel-spatial feature refinement in the encoder, RMSDC performs compact multi-scale contextual aggregation at the bottleneck, and the decoder is intentionally kept lightweight. This stage-specific organization forms the basis of the asymmetric encoder-decoder design investigated in this study.

## 3 Methodology

### 3.1 Overall architecture of the proposed network

Figure 1 presents the overall architecture of the proposed HCSA-RMSDC U-Net. The network follows an asymmetric encoder-decoder organization based on the standard U-Net backbone. HCSA is incorporated into the encoder stages for progressive feature refinement, while RMSDC is positioned at the bottleneck for multi-scale contextual aggregation. The decoder retains bilinear upsampling, skip-feature concatenation, and standard double-convolution blocks without additional HCSA or RMSDC modules. A final 1×1 convolution produces the single-channel output logits.

**Figure 1:**
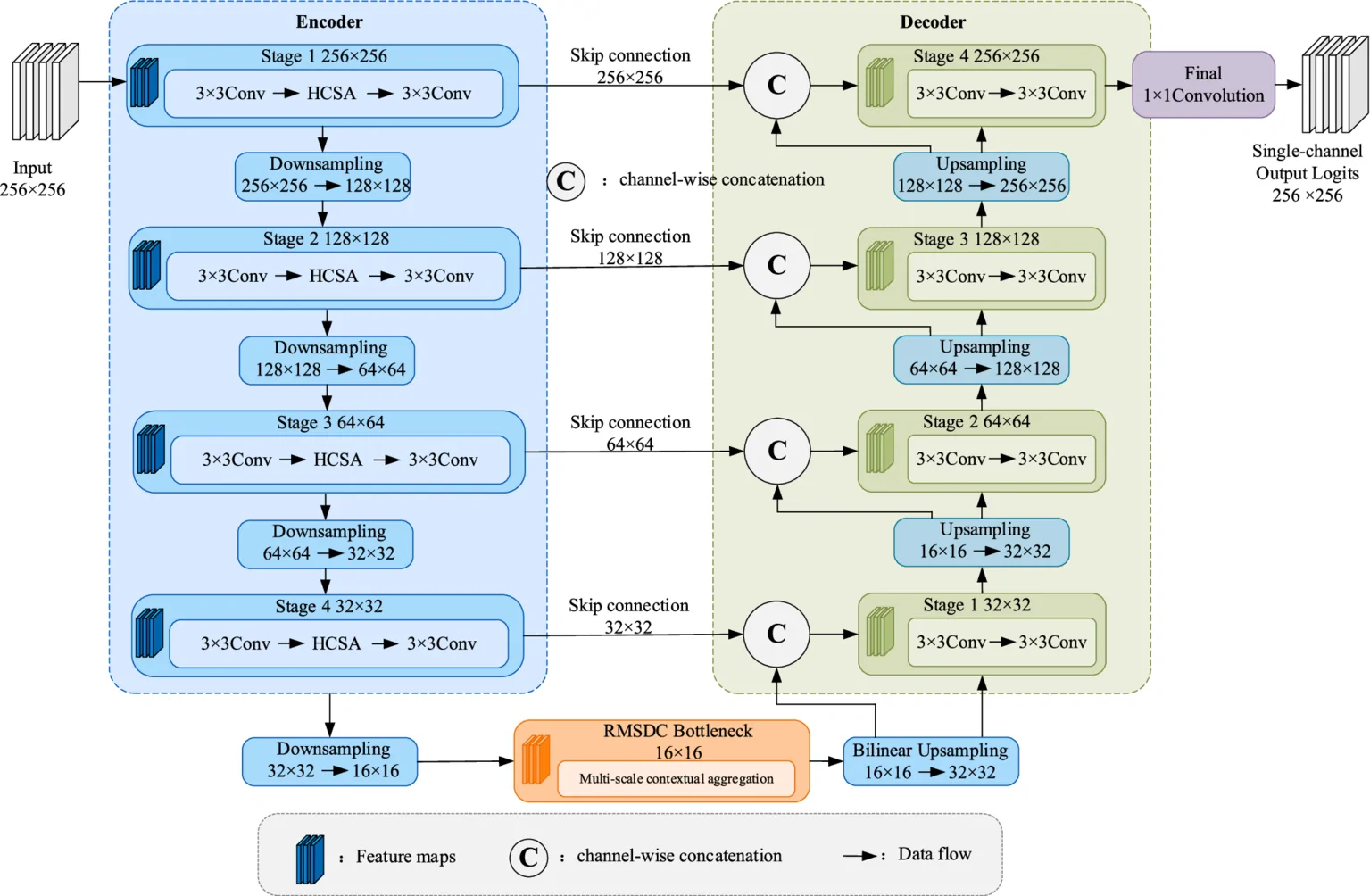
Overall architecture of the proposed HCSA-RMSDC U-Net. The network consists of four HCSA-enhanced encoder stages, an RMSDC bottleneck, and a lightweight decoder. The decoder performs bilinear upsampling, skip-feature concatenation, and standard double-convolution operations. The final 1 × 1 convolution produces single-channel output logits. C denotes channel-wise feature concatenation.

The overall experimental procedure consists of dataset preparation, preprocessing, model training, validation-based checkpoint selection, and final testing. After dataset partitioning and preprocessing, the training subset is used to optimize the proposed network, while the validation subset is used to monitor model performance and select the checkpoint with the highest validation Dice score. The selected checkpoint is subsequently evaluated on the held-out test set. During testing, the network output logits are converted to probability maps using the sigmoid function and binarized at a threshold of 0.5, after which Dice, mIoU, Precision, and HD95 are calculated. Detailed descriptions of the dataset partitioning, preprocessing, training settings, and evaluation metrics are provided in Section 4.

### 3.2 Hybrid channel–spatial attention module

As shown in Figure 2, a hybrid attention mechanism is designed for brain tumor segmentation. Two parallel branches—channel attention and spatial attention—are employed, and are combined with learnable adaptive fusion weights. In the channel attention branch, global average pooling and a multilayer perceptron are utilized to adaptively recalibrate feature channels, so that effective semantic features relevant to the tumor segmentation task are highlighted, while redundant or interfering channel responses are suppressed. In the spatial attention branch, depthwise strip convolutions along the horizontal and vertical directions are adopted to enhance long-range spatial correlations in both orientations, by which contextual associations between tumor regions and their surrounding tissues are strengthened. Subsequently, a weighted fusion of the outputs from the channel and spatial attention branches is performed via learnable adaptive fusion weights, enabling the network to dynamically adjust the importance of the two types of attention information based on the input features. Finally, the original features are recalibrated using the fused attention weights.

**Figure 2:**
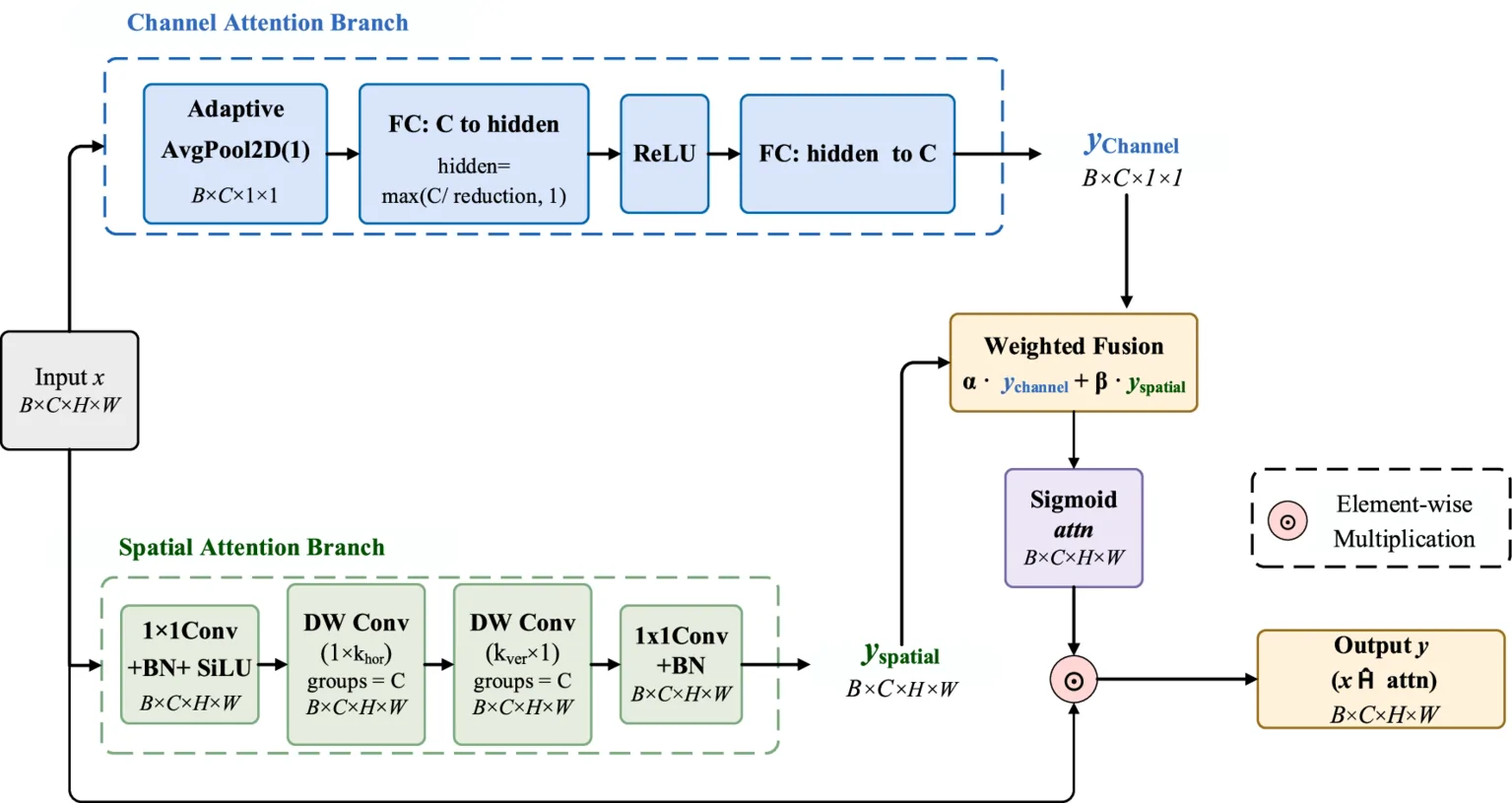
HCSA attention mechanism.

Within the U-Net architecture, the HCSA module is placed between the two 3 × 3 convolutions in each double-convolution block of the encoder. Specifically, the input features are first passed through the first convolution for local feature extraction, then subjected to feature screening by the HCSA module, and are finally fed into the second convolution for further fusion. With this configuration, tumor-related feature responses are progressively enhanced at each encoder stage by the attention mechanism, while the interference of irrelevant background information on subsequent feature extraction is effectively mitigated.

Let the input feature map be $X\in {\mathbb{R}}^{B\times C\times H\times W}$ , where B, C, H, and W denote the batch size, number of channels, height, and width, respectively. To capture global response information across channels, global average pooling is first applied to the input feature, by which the spatial dimensions are compressed. The global descriptor for the c-th channel is thereby obtained, as expressed in Equation 1:

$$z_{c}=\frac{1}{H\times W}\sum_{i=1}^{H}\sum_{j=1}^{W}X_{c}(i,j),\;\;c=1,2,\ldots ,C$$ (1)

Here, $X_{c}(i,j)$ denotes the feature value of the input X at the c-th channel and the (i,j) spatial position, and $z_{c}$ represents the corresponding global response for the c-th channel. The global descriptor vector for all channels is denoted as $z=[z_{1},z_{2},\ldots ,z_{c}]$ , which is subsequently fed into a two-layer fully connected network to further model the dependencies among channels, as shown in Equation 2:

$$Y_{\text{channel}}=W_{2}\delta (W_{1}z)$$ (2)

where $W_{1}$ and $W_{2}$ denote the weight matrices of the two fully connected layers, respectively, δ(·) denotes the ReLU activation function; and $Y_{\text{channel}}\in {\mathbb{R}}^{B\times C\times 1\times 1}$ denotes the resulting channel-attention representation. The singleton spatial dimensions of $Y_{\text{channel}}$ are broadcast over the height and width dimensions when it is subsequently fused with the spatial-attention representation.

In the spatial attention branch, inter-channel information is first fused via a 1 × 1 convolution applied to the input features, through which feature representation is enhanced while the spatial dimensions of the feature map are preserved, as shown in Equation 3:

$$S_{0}=\phi (\mathrm{BN}({\text{Conv}}_{1\times 1}(X)))$$ (3)

Where $\mathrm{Con}v_{1\times 1}$ denotes the 1 × 1 convolution operation, BN denotes batch normalization, and ϕ(⋅) denotes the SiLU activation function. Subsequently, to enlarge the receptive field of the feature map, depthwise strip convolutions are employed along both horizontal and vertical directions for spatial contextual modeling, as formulated in Equations 4, 5:

$$S_{\mathrm{hor}}={\text{DWConv}}_{1\times k_{\mathrm{hor}}}(S_{0})$$ (4)

$$S_{\mathrm{ver}}={\text{DWConv}}_{k_{\mathrm{ver}}\times 1}(S_{\mathrm{hor}})$$ (5)

In Equations 4, 5, DWConv denotes the depthwise convolution operation, $k_{\mathrm{hor}}$ and $k_{\mathrm{ver}}$ represent the kernel lengths of the horizontal and vertical strip convolutions, respectively. Specifically, $1\times k_{\mathrm{hor}}$ represents the horizontal strip convolution that expands along the width dimension, while $k_{\mathrm{ver}}\times 1$ represents the vertical strip convolution that expands along the height dimension. In the experimental setup, both $k_{\mathrm{hor}}$ and $k_{\mathrm{ver}}$ are set to 11. Compared with the standard 3 × 3 convolution kernel, a broader range of spatial contextual information can be captured through the use of strip convolutions.

Subsequently, after spatial context modeling has been performed along the horizontal and vertical directions, a 1 × 1 convolution is further applied so that the spatial features are integrated, and the spatial attention features are thereby obtained, as expressed in Equation 6:

$$Y_{\text{spatial}}=\mathrm{BN}({\text{Conv}}_{1\times 1}(S_{\mathrm{ver}}))$$ (6)

The operations in the spatial branch preserve the spatial resolution, and the resulting spatial-attention representation satisfies $Y_{\text{spatial}}\in {\mathbb{R}}^{B\times C\times H\times W}$ . These spatial attention features facilitate the extraction of contextual information relevant to tumor regions.

Subsequently, in order to fully fuse the channel and spatial attention, two learnable parameters, α and β, are introduced, by which adaptive weighted fusion of the two attention features is performed. The final attention weight map is then generated through a Sigmoid function, as formulated in Equation 7:

$$A=\sigma (\alpha Y_{\text{channel}}+\beta Y_{\text{spatial}})$$ (7)

where σ(⋅) denotes the Sigmoid activation function and $A\in {\mathbb{R}}^{B\times C\times H\times W}$ denotes the final attention weight map, and α and β are trainable scalar fusion coefficients initialized to 1.0. During training, α and β are optimized jointly with the remaining network parameters to adaptively regulate the relative contributions of channel and spatial attention.

Finally, an element-wise multiplication is performed between the attention weight map and the input features, and the output of the HCSA module is thereby obtained, as formulated in Equation 8:

Y=X⊙A (8)

where element-wise multiplication is denoted by ⊙ , and the output feature map of the HCSA module is represented by $Y\in {\mathbb{R}}^{B\times C\times H\times W}$ .

In summary, effective semantic channels relevant to tumor segmentation are selectively screened by the HCSA module through channel attention, and spatial context modeling is enhanced via horizontal and vertical strip convolutions. Consequently, the encoder is enabled to focus more on tumor regions and their surrounding tissue information during feature extraction, by which more discriminative encoded features are provided for subsequent multi-scale context modeling at the bottleneck and spatial recovery during the decoding stage.

The distinguishing characteristic of HCSA lies in the joint organization of these components rather than in any individual attention operation. In particular, channel and directional spatial representations are generated in parallel, the spatial branch preserves directional contextual information through horizontal and vertical depthwise strip convolutions, and the two representations are adaptively fused through learnable coefficients before feature recalibration. Moreover, HCSA is inserted within the encoder double-convolution blocks so that feature refinement is performed progressively during encoding, rather than only at skip connections or decoder-side fusion stages.

### 3.3 Residual multi-scale dilated convolution module

In brain tumor segmentation tasks, substantial variations in shape and size are exhibited by tumor regions, and blurred boundaries with low contrast against surrounding brain tissues are frequently observed. As a result, critical details may be lost or contextual information may be neglected by the model during feature extraction. With standard convolutions, operations are restricted to fixed local neighborhoods; therefore, when the spatial extent of tumor regions varies considerably, simultaneous characterization of small-scale local structures and large-range contextual relationships cannot be achieved by a single receptive field. As downsampling is progressively performed by the U-Net encoder on the feature maps, the deep features become semantically richer but spatially more compressed. Consequently, inadequate modeling of tumor structural information across varying ranges may result from relying solely on consecutive standard convolutions at the bottleneck.

To address the aforementioned issues, a residual multi-scale dilated convolution (RMSDC) module is designed and introduced at the bottleneck of the U-Net, which is specifically positioned after the fourth downsampling stage and before the first upsampling stage. The high-level semantic features produced by the encoder are enhanced through this module, and the refined features are subsequently fed into the decoder. The RMSDC module is composed of one identity branch and three dilated convolution branches, which together constitute a parallel context modeling structure; through the use of varying dilation rates, features with distinct receptive fields are obtained. In addition, a learnable residual coefficient is adopted to regulate the injection strength of the enhanced information. The overall architecture of the module is illustrated in Figure 3.

**Figure 3:**
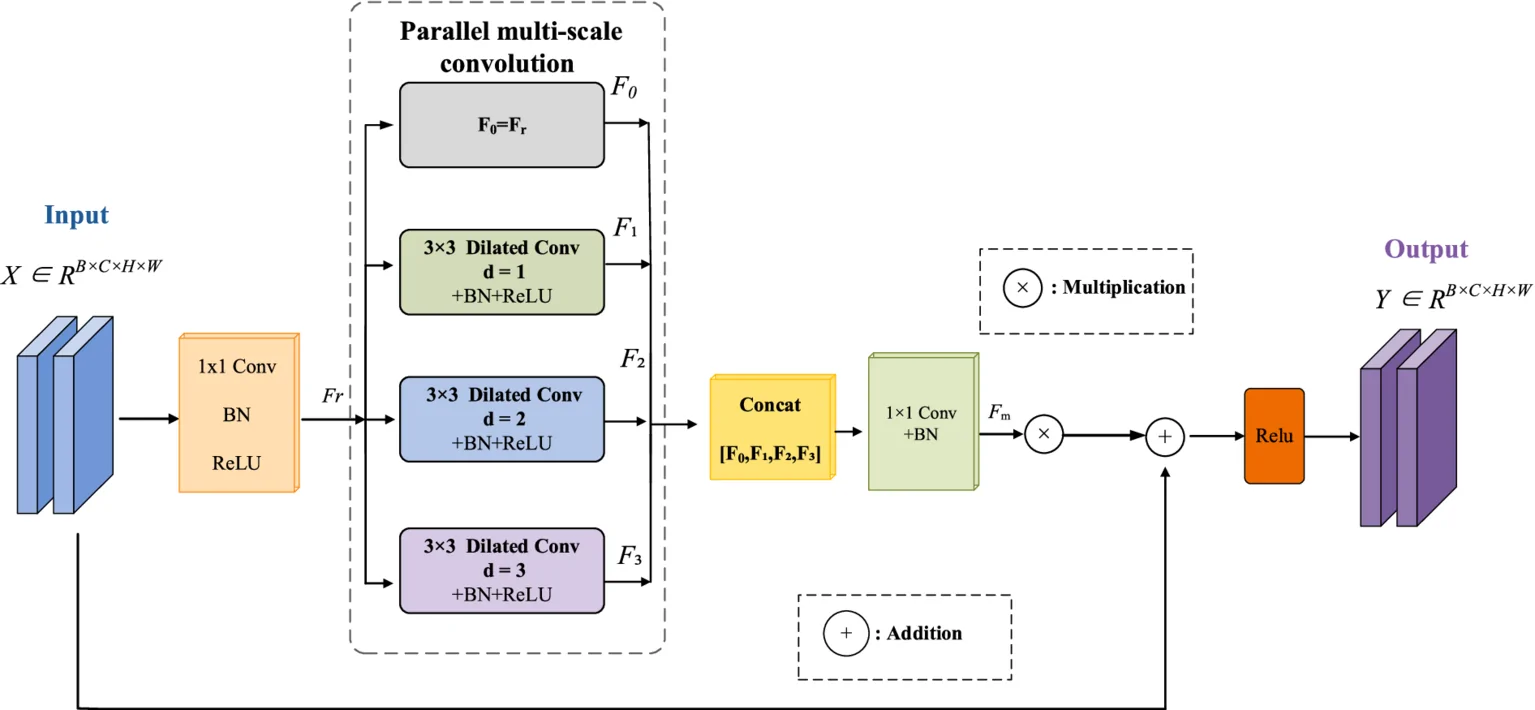
Residual multi-scale dilated convolution module.

Given the input features $X\in {\mathbb{R}}^{B\times C\times H\times W}$ , a 1 × 1 convolution is first applied so that the channel dimension is compressed, which is followed by batch normalization and a ReLU activation function. The reduced-dimensional features $F_{r}$ are thereby yielded, as formulated in Equation 9:

$$F_{r}=\delta (\mathrm{BN}(\mathrm{Con}v_{1\times 1}(X)))$$ (9)

where the ReLU activation function is denoted by δ(.) , and batch normalization is denoted by BN(.) .

Subsequently, after channel compression has been performed, the reduced-dimensional features $F_{r}$ are concurrently fed into four parallel branches. The first of these branches is configured as the identity branch, through which no additional convolutional transformation is applied to the input. The output representation of this branch is obtained as shown in Equation 10:

$$F_{0}=F_{r}$$ (10)

The identity branch is primarily employed to preserve the local semantic information that is already present in the reduced-dimensional features. Since interval sampling is adopted by dilated convolutions, the continuous local responses could be weakened if all branches were implemented with dilated convolutions. Through the direct preservation of the original features $F_{r}$ , baseline features that have not been subjected to dilated sampling are provided by the identity branch for subsequent feature fusion.

Subsequently, 3 × 3 dilated convolutions with dilation rates of 1, 2, and 3 are, respectively, employed in the remaining three branches, with each branch being sequentially followed by batch normalization and ReLU activation. The output of the i-th dilated convolution branch is expressed as shown in Equation 11:

$$F_{i}=\delta (\mathrm{BN}(\mathrm{Con}v_{3\times 3}^{d_{i}}(F_{r}))),\;d_{i}\in \{1,2,3\},\;i=1,2,3,$$ (11)

where $\mathrm{Con}v_{3\times 3}^{d_{i}}(.)$ denotes the dilation rate of the 3 × 3 dilated convolution operation with a dilation rate of $d_{i}$ . Dilated convolution enlarges the effective receptive field by introducing gaps between sampling positions in the convolution kernel, without reducing the spatial resolution of the feature maps. The three dilated convolution branches extract bottleneck features from different spatial ranges: the branch with a dilation rate of 1 focuses on local structures in the adjacent region, while the branches with dilation rates of 2 and 3 progressively expand the receptive range to model broader contextual relationships between tumor regions and surrounding tissues.

Subsequently, after parallel context modeling has been performed, the outputs from the identity branch and the three dilated convolution branches are concatenated along the channel dimension. A 1 × 1 convolution kernel is then applied to the concatenated features so that cross-channel fusion is carried out, which is followed by batch normalization for feature distribution adjustment. The fused features $F_{m}$ are thereby obtained, as expressed in Equations 12, 13:

$$F_{\mathrm{cat}}=\text{Concat}(F_{0},F_{1},F_{2},F_{3})$$ (12)

$$F_{m}=\mathrm{BN}(\mathrm{Con}v_{1\times 1}(F_{\mathrm{cat}}))$$ (13)

where the total number of channels after concatenation is restored to the original channel count C. The information extracted by each branch is preserved in a relatively complete manner through channel concatenation, by which the fusion layer is enabled to autonomously learn the combinatorial relationships among features originating from different receptive fields.

Finally, a channel-wise learnable residual coefficient $\gamma \in {\mathbb{R}}^{1\times C\times 1\times 1}$ is introduced to regulate the contribution of the fused multi-scale feature $F_{m}$ to the original bottleneck feature X . All elements of γ are initialized to $10^{-3}$ and are subsequently optimized jointly with the remaining network parameters. During element-wise multiplication, γ is broadcast over the batch and spatial dimensions. The fused feature $F_{m}$ is first multiplied element-wise by γ and is then added to the module input X . The resulting sum is subsequently passed through a ReLU activation function to obtain the final output, as formulated in Equation 14:

$$Y=\delta (X+\gamma ⊙F_{m})$$ (14)

where the symbol ⊙ denotes element-wise multiplication, and $Y\in {\mathbb{R}}^{B\times C\times H\times W}$ denotes the final output. Consequently, the spatial dimensions and the number of channels of the feature map are preserved by the RMSDC module, which can therefore be directly connected to the subsequent first upsampling module.

The distinctive design of RMSDC lies in the coordinated use of an identity-preserving branch, compact multi-rate dilated convolutions, feature fusion, and learnable residual scaling. In contrast to simply replacing standard convolution with dilated convolution, RMSDC retains the original local semantic representation while introducing complementary contextual information from multiple receptive fields. The module is restricted to the bottleneck so that multi-scale enhancement is performed on high-level semantic features before decoding, while additional complexity is avoided in the decoder.

## 4 Experiments

### 4.1 Datasets

In this study, two publicly available brain tumor MRI datasets were employed for model evaluation: the BRISC 2025 dataset and the Figshare brain tumor dataset.

The BRISC2025 dataset (Fateh et al., 2026) is an expert-annotated brain tumor MRI dataset developed for both segmentation and classification tasks. It contains two-dimensional contrast-enhanced T1-weighted MRI images acquired in axial, coronal, and sagittal planes. The segmentation subset consists of 4,793 MRI images with corresponding pixel-wise tumor masks, and the annotations were reviewed by medical experts to improve label and mask quality. In this study, only the segmentation subset was used. Since patient identifiers are not provided in the publicly released BRISC2025 dataset, patient-level partitioning could not be performed. Therefore, the official training partition was retained as the training set, while the official test partition was further divided into validation and test subsets at the image level using class-stratified random sampling with a 1:1 ratio and a fixed random seed of 42.

The publicly available Figshare brain tumor dataset was constructed by Cheng et al. It comprises 3,064 T1-weighted contrast-enhanced MRI images acquired from 233 patients. The images are categorized into three tumor types, consisting of 1,426 glioma, 708 meningioma, and 930 pituitary tumor cases. Since patient identifiers are available for this dataset, the data were partitioned at the patient level into training, validation, and test subsets with a ratio of 8:1:1. All images from the same patient were assigned exclusively to a single subset, thereby preventing patient overlap among the three subsets. The resulting partition was fixed in advance and reused consistently for all experiments. This dataset has been extensively used in brain tumor segmentation and classification studies.

### 4.2 Experimental details and evaluation metrics

All experiments were implemented using the PyTorch deep learning framework and were conducted on a single NVIDIA RTX 4080 s GPU for both training and validation. The network was optimized using the AdamW optimizer, with an initial learning rate of $1\times 10^{-4}$ and a weight decay coefficient of $1\times 10^{-4}$ . The loss function was defined as BCEDiceLoss, which is a weighted combination of binary cross-entropy loss and Dice loss. Training was carried out for 100 epochs, with a batch size of 4 being adopted.

Before being fed into the network, all MRI images were converted to grayscale and resized to 256×256 pixels using bilinear interpolation. The corresponding segmentation masks were resized to the same spatial resolution using nearest-neighbor interpolation and were binarized using a threshold of 127. Image intensities were scaled to [0,1] by division by 255. During training, random horizontal and vertical flips were independently applied with a probability of 0.5, followed by a random rotation selected from $0^{\circ }$ , $90^{\circ }$ , $180^{\circ }$ , and $270^{\circ }$ . No stochastic data augmentation was applied to the validation or test sets.

The same fixed dataset partitions were used for all model configurations. No explicit global random seed was imposed during model training in the original experiments; therefore, stochastic operations such as parameter initialization, mini-batch shuffling, and training-time augmentation were governed by the runtime random state. During training, model performance was monitored on the validation set, and the checkpoint achieving the highest validation Dice score was selected for final testing. Each model/configuration was trained once, and the reported quantitative results therefore correspond to a single training run rather than averages over multiple independent runs.

In this study, four evaluation metrics were employed to assess the performance of the model, including the Dice similarity coefficient (Dice), mean Intersection over Union (mIoU), 95% Hausdorff distance (HD95), and Precision. The mathematical definitions of Dice, foreground IoU, background IoU, mIoU, and Precision are presented in Equations 15–19, respectively.

For binary segmentation, Dice was calculated for the foreground class as

$$D\mathrm{ice}=\frac{2\mathrm{TP}}{2\mathrm{TP}+\mathrm{FP}+\mathrm{FN}+\varepsilon }$$ (15)

where $\varepsilon =10^{-8}$ is a small constant used to avoid division by zero.

The foreground and background IoUs were calculated as

$$\mathrm{Io}U_{\mathrm{fg}}=\frac{\mathrm{TP}}{\mathrm{TP}+\mathrm{FP}+\mathrm{FN}+\varepsilon }$$ (16)

and

$$\mathrm{Io}U_{\mathrm{bg}}=\frac{\mathrm{TN}}{\mathrm{TN}+\mathrm{FP}+\mathrm{FN}+\varepsilon }$$ (17)

respectively. The mIoU was then defined as the arithmetic mean of the foreground and background IoUs:

$$\text{mIoU}=\frac{\mathrm{Io}U_{\mathrm{fg}}+\mathrm{Io}U_{\mathrm{bg}}}{2}$$ (18)

Precision was calculated as

$$\mathrm{Pr}\text{ecision}=\frac{\mathrm{TP}}{\mathrm{TP}+\mathrm{FP}+\varepsilon }$$ (19)

where TP , TN , FP and FN denote the numbers of true-positive, true-negative, false-positive, and false-negative pixels, respectively. HD95 was computed as the 95th percentile of the bidirectional boundary distances between the predicted and ground-truth tumor masks using MedPy, with the voxel spacing set to 0.49. When both masks contained foreground pixels, HD95 was computed directly. When only one mask was empty, the image-diagonal distance was used as a penalty and capped at 100; when both masks were empty, HD95 was set to 0. The predicted probability maps were converted into binary masks using a threshold of 0.5 after the sigmoid operation. During testing, all evaluation metrics were calculated independently for each test image and subsequently averaged over the entire test set using image-wise macro averaging. All ground-truth masks in the Figshare and BRISC2025 test sets were verified to contain foreground tumor regions; therefore, no empty ground-truth masks occurred during evaluation. Empty predictions, if present, were retained rather than excluded. In such cases, foreground Dice, foreground IoU, and Precision evaluate to 0 according to the definitions above, while mIoU remains the arithmetic mean of the foreground and background IoUs. For HD95, when only one of the prediction and ground-truth masks was empty, the image-diagonal penalty described above was applied. All test images were included in the final image-wise macro averaging (Table 1).

**Table 1:** Experimental hyperparameters.

Hyperparameter	Value
Optimizer	AdamW
Weight decay	0.0001
Learning rate	0.0001
Learning rate scheduler	ReduceLROnPlateau
Input image size	256 × 256
Batch size	4

During training, a combined loss function, BCEDiceLoss, was adopted to address the severe class imbalance between foreground and background pixels in medical image segmentation. This loss function integrates binary cross-entropy (BCE) loss with Dice loss, by which the complementary advantages of both are leveraged. Specifically, stable pixel-wise gradients are provided by BCE loss, whereas direct optimization of the overlap between predicted and ground-truth segmentation regions is achieved through Dice loss. The BCE loss, Dice loss, and final combined loss are formulated in Equations 20–22, respectively. For the binary segmentation task, let N denote the total number of pixels in the image, $g_{i}\in \{0,1\}$ denote the ground-truth label of pixel i, and $p_{i}\in [0,1]$ denote the predicted probability of the foreground for pixel i. The binary cross-entropy loss is defined as follows:

$$L_{\mathrm{BCE}}=-\frac{1}{N}\sum_{i=1}^{N}[g_{i}\log(p_{i})+(1-g_{i})\log(1-p_{i})]$$ (20)

In the context of continuous probability outputs, a smoothing term is introduced by the Dice loss to prevent the denominator from becoming zero, and the loss function is consequently defined as follows:

$$L_{\text{Dice}}=1-\frac{2\sum_{i=1}^{N}p_{i}g_{i}+\varepsilon }{\sum_{i=1}^{N}p_{i}+\sum_{i=1}^{N}g_{i}+\varepsilon }$$ (21)

The final combined loss function is defined as a weighted sum of the two loss components, which is formulated as follows:

$$L_{\text{total}}=0.5\times L_{\mathrm{BCE}}+L_{\text{Dice}}$$ (22)

### 4.3 Comparative experiment

As shown in Table 2, the proposed HCSA-RMSDC U-Net achieved a Dice score of 82.84%, an mIoU of 86.59%, a Precision of 82.81%, and an HD95 of 4.778 on the Figshare dataset. Among the evaluated methods, the proposed model obtained the highest Dice, mIoU, and Precision scores, as well as the lowest HD95. Compared with CBAMUNet, which achieved the highest Dice score among the competing methods, the proposed model improved Dice and mIoU by 0.94 and 0.25 percentage points, respectively, while reducing HD95 from 5.363 to 4.778. These results indicate that the proposed method achieves favorable segmentation overlap and boundary-distance performance on the Figshare dataset.

**Table 2:** Quantitative analysis results on the Figshare dataset.

Methods	Figshare			
	Dice↑	mIoU↑	Prec↑	HD95↓
UNet (Ronneberger et al., 2015)	78.85	84.60	79.95	7.339
UNet++ (Zhou et al., 2018)	79.36	84.76	80.23	7.856
AttentionUNet (Oktay et al., 2018)	78.32	84.21	78.92	8.417
CBAMUNet (Song et al., 2025)	81.90	86.34	82.37	5.363
CMUNet (Tang et al., 2023)	80.35	85.24	81.20	5.417
DeepLab (Chen et al., 2018)	78.21	84.06	79.94	7.036
MKUNet (Rahman and Marculescu, 2025)	80.78	85.31	81.75	5.442
MultiResUNet (Ibtehaz and Rahman, 2020)	78.18	84.32	79.17	8.288
UKAN (Li et al., 2025)	80.48	85.49	81.16	6.496
Ours	82.84	86.59	82.81	4.778

The quantitative results on the BRISC2025 dataset are presented in Table 3. The proposed method achieved a Dice score of 87.39%, an mIoU of 90.20%, a Precision of 88.25%, and an HD95 of 3.586. Compared with the competing methods, the proposed model obtained the highest Dice and mIoU scores and the lowest HD95. In particular, compared with CBAMUNet, which achieved a Dice score of 87.18%, the proposed model improved Dice by 0.21 percentage points and mIoU by 0.36 percentage points, while reducing HD95 from 4.338 to 3.586. However, CBAMUNet achieved a slightly higher Precision of 88.28%, compared with 88.25% for the proposed model. These findings suggest that the proposed architecture provides favorable overall segmentation performance on the BRISC2025 dataset, particularly in terms of region overlap and boundary-distance measures.

**Table 3:** Quantitative analysis results on the BRISC 2025 dataset.

Methods	BRISC 2025			
	Dice↑	mIoU↑	Prec↑	HD95↓
UNet (Ronneberger et al., 2015)	86.95	89.88	87.69	5.210
UNet++ (Zhou et al., 2018)	87.15	89.95	87.75	5.109
AttentionUNet (Oktay et al., 2018)	86.70	89.64	86.72	5.412
CBAMUNet (Song et al., 2025)	87.18	89.84	88.28	4.338
MKUNet (Rahman and Marculescu, 2025)	86.00	89.07	86.67	4.486
CMUNet (Tang et al., 2023)	86.94	89.98	87.71	3.661
MultiResUNet (Ibtehaz and Rahman, 2020)	86.42	89.38	85.15	5.485
SwinUNet (Li, 2026)	78.61	83.97	80.83	8.431
Ours	87.39	90.20	88.25	3.586

To provide a more comprehensive view of the proposed model’s performance across training, validation, and testing, Table 4 summarizes the recorded segmentation metrics at the three experimental stages for both datasets. The training and validation metrics correspond to the epoch achieving the highest validation Dice score, while the testing metrics were obtained by evaluating the selected checkpoint on the held-out test set. Specifically, the proposed model achieved training and testing Dice scores of 86.31 and 82.84% on the Figshare dataset, respectively. On the BRISC2025 dataset, the corresponding Dice scores were 90.29 and 87.39%. The associated mIoU and Precision values are also presented in Table 4.

**Table 4:** Training, validation, and testing performance of the proposed HCSA-RMSDC U-Net on the Figshare and BRISC2025 datasets.

Dataset	Stage	Dice↑	mIoU↑	Precision↑
Figshare	Training	86.31	88.12	86.58
	Validation	85.61	87.70	86.75
	Testing	82.84	86.59	82.81
BRISC2025	Training	90.29	91.33	91.13
	Validation	88.75	90.31	88.63
	Testing	87.39	90.20	88.25

### 4.4 Visualization results

To further assess the segmentation quality of the proposed model, visual comparisons of the segmentation results are presented. Representative segmentation results obtained by the proposed model and four selected competing methods are presented for each of the Figshare and BRISC2025 datasets. The qualitative results show that, in the representative cases presented, the proposed model generally produces segmentation masks that more closely align with the ground-truth annotations, particularly in the delineation of tumor regions (Figures 4, 5).

**Figure 4:**
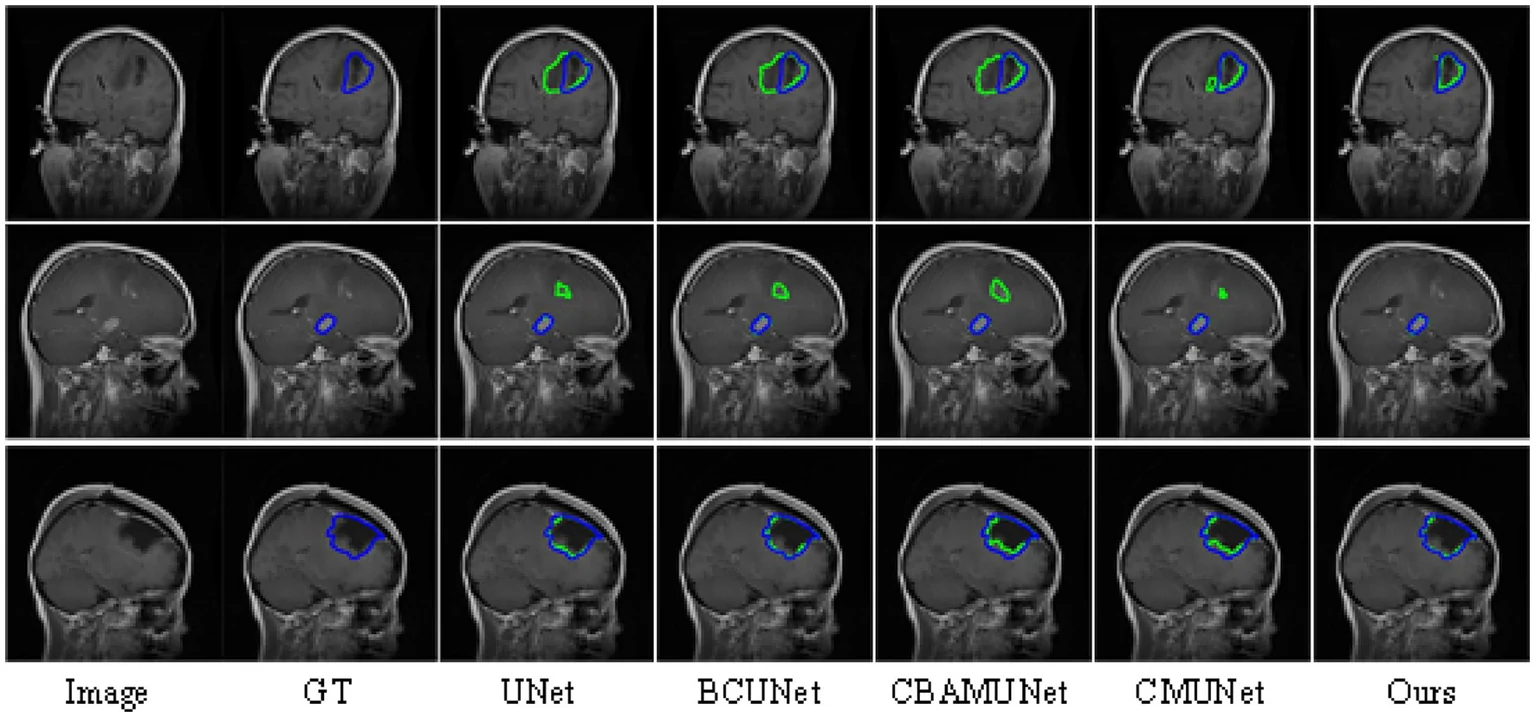
Visual comparison of segmentation results from different models on the Figshare dataset. Blue and green denote the ground truth and the predicted segmentation, respectively.

**Figure 5:**
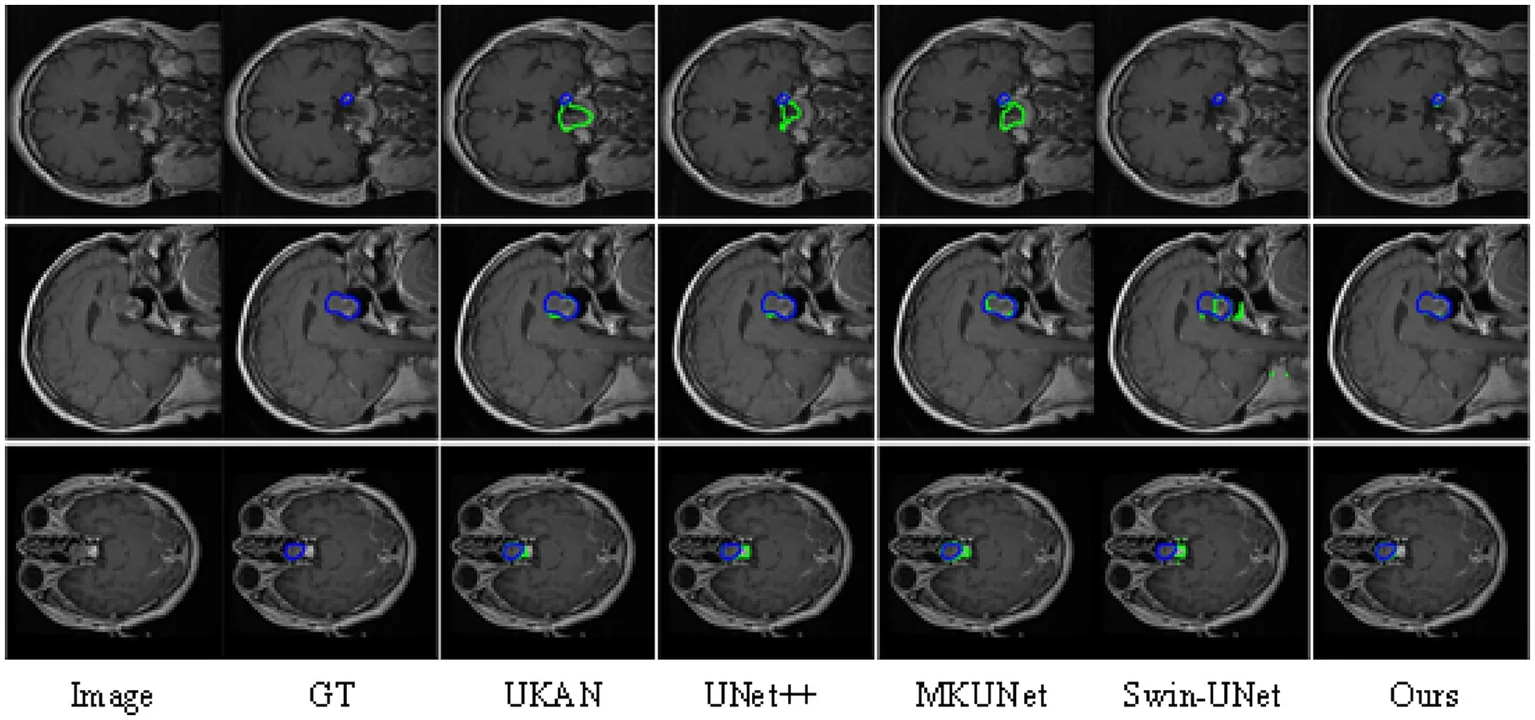
Visual comparison of segmentation results from different models on the BRISC 2025 dataset. Blue and green denote the ground truth and the predicted segmentation, respectively.

### 4.5 Ablation studies

In this section, a series of ablation experiments are conducted on the Figshare dataset to evaluate the contributions of the key components of the proposed model. The effects of HCSA and RMSDC are examined individually and in combination using the U-Net baseline, as summarized in Table 5. Both individual modules improve the segmentation performance over the baseline, while their combined use achieves the best overall results among the evaluated configurations. Overall, the combined HCSA-RMSDC configuration achieves the best results among the evaluated variants, improving Dice and mIoU by 3.99 and 1.99 percentage points, respectively, compared with the U-Net baseline. These results demonstrate the individual contributions of HCSA and RMSDC and the performance obtained when the two modules are used together.

**Table 5:** Ablation experiments on the impact of key modules on the Figshare dataset.

Key modules		Evaluation metrics			
RMSDC	HCSA	Dice↑	mIoU↑	Prec↑	HD95↓
×	×	78.85	84.60	79.95	7.339
√	×	79.96	85.10	81.04	5.799
×	√	82.40	86.39	82.46	5.131
√	√	82.84	86.59	82.81	4.778

To further evaluate the heavy-encoder/light-decoder design, a symmetric counterpart of the proposed architecture was constructed. The encoder and RMSDC bottleneck were kept identical to those of the proposed model, while HCSA was additionally introduced into the corresponding decoder blocks. All other architectural and training settings were unchanged. As shown in Table 6, the symmetric counterpart achieved a Dice score of 81.63%, mIoU of 86.22%, Precision of 82.84%, and HD95 of 5.018, whereas the proposed asymmetric model achieved 82.84%, 86.59%, 82.81%, and 4.778, respectively. Although the symmetric model achieved a marginally higher Precision, the proposed asymmetric model obtained better Dice, mIoU, and HD95 while requiring fewer parameters and lower computational cost. Specifically, compared with the symmetric counterpart, the proposed asymmetric model reduced the parameter count from 19.65 M to 18.84 M and the computational cost from 44.74 GMACs to 42.42 GMACs. These results suggest that duplicating HCSA in the decoder does not provide a commensurate performance benefit under the evaluated setting, while supporting the lightweight decoder as a more favorable performance–complexity trade-off (Table 7).

**Table 6:** Ablation and computational comparison of the asymmetric and symmetric architectural designs on the Figshare dataset.

Architecture	Encoder HCSA	Bottleneck RMSDC	Decoder HCSA	Params(M)	GMACs	Dice_↑_	mIoU_↑_	Prec_↑_	HD95_↓_
UNet	×	×	×	17.26	39.90	78.85	84.60	79.95	7.339
Symmetric counterpart	√	√	√	19.65	44.74	81.63	86.22	82.84	5.018
Ours(asymmetric)	√	√	×	18.84	42.42	82.84	86.59	82.81	4.778

**Table 7:** Internal design ablation of HCSA and RMSDC on the Figshare dataset.

Module	Variant	Dice_↑_	mIoU_↑_	Prec_↑_	HD95_↓_
HCSA	Fixed fusion	80.83	85.63	82.37	6.229
HCSA	Learnable fusion	82.40	86.39	82.46	5.131
RMSDC	Uniform dilation(1,1,1)	80.20	85.28	81.28	6.941
RMSDC	Multi-rate dilation(1,2,3)	79.96	85.10	81.04	5.799

To further examine the internal design choices of HCSA and RMSDC, two controlled variants were evaluated on the Figshare dataset. For HCSA, replacing the learnable fusion coefficients with fixed summation reduced Dice from 82.40 to 80.83%, mIoU from 86.39 to 85.63%, and increased HD95 from 5.131 to 6.229. These results support the benefit of learnable fusion for adaptively combining channel and spatial responses.

For RMSDC, replacing the multi-rate dilation configuration (1,2,3) with uniform dilation (1,1,1) resulted in slightly higher Dice, mIoU, and Precision, but increased HD95 from 5.799 to 6.941. This result suggests that the multi-rate dilation configuration may provide an advantage in boundary-distance performance, as reflected by the lower HD95, rather than uniformly improving all overlap-based metrics under the evaluated setting.

## 5 Conclusion

In this paper, an HCSA-RMSDC U-Net is designed to address two major challenges in brain tumor MRI segmentation: blurred boundaries and substantial scale variations. The standard U-Net is adopted as the backbone of the designed network. In the encoder, a hybrid channel–spatial attention (HCSA) module is embedded, by which long-range dependencies along the horizontal and vertical directions are captured via strip-wise decomposed convolutions, thereby enhancing directional spatial context modeling and encoder-side feature representation. At the bottleneck, a residual multi-scale dilated convolution (RMSDC) module is introduced to capture multi-scale contextual information through parallel branches with varying dilation rates, by which the network’s adaptability to tumors of different sizes is improved. Through the stage-specific integration of encoder enhancement and bottleneck receptive-field expansion, the two modules combine feature discrimination with multi-scale aggregation. Extensive experiments were conducted on two publicly available brain tumor MRI datasets—BRISC 2025 and Figshare. The experimental results show that the combined use of HCSA and RMSDC improves the overall segmentation performance. Nevertheless, certain limitations are still associated with this study. Specifically, the current method was validated only on 2D slices, and thus the 3D spatial contextual information inherent in MRI has not been fully exploited. Future work will explore the extension of the designed model to a 3D version.

## Data Availability

Publicly available datasets were analyzed in this study. This data can be found at: https://www.kaggle.com/datasets/briscdataset/brisc2025 and https://figshare.com/articles/dataset/brain_tumor_dataset/1512427.
